# Sensitivity Enhancement in Si Nanophotonic Waveguides Used for Refractive Index Sensing

**DOI:** 10.3390/s16030324

**Published:** 2016-03-03

**Authors:** Yaocheng Shi, Ke Ma, Daoxin Dai

**Affiliations:** Centre for Optical and Electromagnetic Research, State Key Laboratory for Modern Optical Instrumentation, Zhejiang Provincial Key Laboratory for Sensing Technologies, Zhejiang University, Zijingang Campus, Hangzhou 310058, China; make@coer-zju.org (K.M.); dxdai@zju.edu.cn (D.D.)

**Keywords:** sensitivity, silicon, nanowire, nanoslot, nanofiber

## Abstract

A comparative study is given for the sensitivity of several typical Si nanophotonic waveguides, including SOI (silicon-on-insulator) nanowires, nanoslot waveguides, suspended Si nanowires, and nanofibers. The cases for gas sensing (*n*_cl_ ~ 1.0) and liquid sensing (*n*_cl_ ~ 1.33) are considered. When using SOI nanowires (with a SiO_2_ buffer layer), the sensitivity for liquid sensing (*S* ~ 0.55) is higher than that for gas sensing (*S* ~ 0.35) due to lower asymmetry in the vertical direction. By using SOI nanoslot waveguides, suspended Si nanowires, and Si nanofibers, one could achieve a higher sensitivity compared to sensing with a free-space beam (*S* = 1.0). The sensitivity for gas sensing is higher than that for liquid sensing due to the higher index-contrast. The waveguide sensitivity of an optimized suspended Si nanowire for gas sensing is as high as 1.5, which is much higher than that of a SOI nanoslot waveguide. Furthermore, the optimal design has very large tolerance to the core width variation due to the fabrication error (∆*w* ~ ±50 nm). In contrast, a Si nanofiber could also give a very high sensitivity (e.g., ~1.43) while the fabrication tolerance is very small (*i.e.*, ∆*w* < ±5 nm). The comparative study shows that suspended Si nanowire is a good choice to achieve ultra-high waveguide sensitivity.

## 1. Introduction

Optical waveguide sensors are paving the way for realizing low-cost, highly sensitive, ultra-compact optical sensors, which are desired for many applications such as biological, environmental and chemical detections [1,2,3,4,5,6,7,8,9]. It is also easy to have a sensor array based on optical waveguides. Usually, the principle of optical waveguide sensors is based on the perturbation of the field of a guided mode caused by optical absorptions, fluorescence or refractive index changes of the measured sample [8]. Among them, refractive index sensors are popular because of their easy realization, and the potential for real-time monitoring with a minimal sample volume. When the concentration of the sample covering on the waveguide surface changes, the effective refractive index of the optical waveguide changes and consequently a phase shift will be introduced. This phase shift could be converted into an intensity change or a frequency shift using interferometers or resonant structures. In past years, people have developed various integrated optical sensors based on different structures and mechanisms, e.g., Mach–Zehnder interferometers (MZI) [1], and high-Q optical microcavities (including microrings/microdisks [2,3,4,5,6,7,8,9,10,11]).

When designing and fabricating an optical sensor, sensitivity is one of the most important figures of merit to consider. Generally speaking, there are two parts contributing to the total sensitivity, *i.e.*, the waveguide sensitivity (S_WG_) and the device sensitivity (S_d_) [11]. The device sensitivity is the ratio of the change in the measured optical parameter (*i.e.*, the resonance wavelength, or the intensity at a specific wavelength) to the change of the effective index. The waveguide sensitivity S_WG_ is defined as the ratio of the effective index change ∆n_eff_ to the change ∆n_s_ of the sample index, *i.e.*, S_WG_ = ∆n_eff_/∆n_s_. In order to improve the sensitivity of an optical sensor, one should improve the device design as well as the waveguide design. The device sensitivity S_d_ mainly depends on the device structure while the waveguide sensitivity S_WG_ depends on the waveguide cross section as well as the refractive index profile. One can optimize the device structure and the waveguide structure separately to maximize the device sensitivity and the waveguide sensitivity, respectively. For example, an ultra-high sensitivity was achieved using the Vernier effect in a dual-ring system [12,13,14], and the waveguide sensitivity can be improved using some special waveguides, which will be discussed in this paper.

Since only the evanescent field (which is a small part of the total guided-modal field) “experiences” the analyzed medium, the sensitivity SWG of a guided mode in an optical waveguide is usually assumed to be smaller or much smaller than that of a free-space beam (S = 1) [15]. This is true when using a conventional strip or rib waveguide with a large core size and a low index-contrast because the evanescent fields are not very strong. For example, the sensitivity for a SiO_2_ or polymer waveguide is usually less than 0.1 [1], which is much smaller than that of a free-space beam. Hollow-core waveguides [16] are developed to obtain a higher sensitivity in the way of guiding light in the low-index sample material using the Bragg-grating effect. However, this also makes the waveguide transmission highly wavelength dependent and furthermore the fabrication is quite complicated.

Recently, Si nanowires have become a favored choice because of their evanescent field enhancement in the cladding region due to the small cross section and the ultra-high index contrast. TM polarization is usually used to have higher sensitivity, S_WG_ ≈ 0.5 [1], which, however, is still less than that of a free-space beam (S = 1). In 2004, a nanoslot waveguide was introduced as a novel guided-wave configuration [17], in which there is a field enhancement in the low-index slot region due to the boundary condition of the perpendicular electrical component. This makes it very attractive to achieve high sensitivity for optical sensing [18,19,20]. For a nanoslot waveguide, the optimized waveguide sensitivity could be as high as 1.0 [19,20], which provides a way to realizing a waveguide sensitivity of more than the sensitivity of a free-space beam (S = 1). In [15], the author gave an analytical analysis for a three-layer slab waveguide and reported a peculiar effect, namely that the waveguide sensitivity can indeed be larger than 1.0 for TM polarization. This means that an optical waveguide sensor can be made with a higher sensitivity than a free-space configuration.

In this paper, we consider the case with three-dimensional nanophotonic waveguides and give a comparative study for the sensitivity of several typical silicon nanophotonic waveguides with a very-high index-contrast, e.g., SOI (silicon-on-insulator) nanowires, SOI nanoslot waveguides, suspended Si nanowires, and Si nanofibers. Our simulation shows that an enhanced sensitivity of about 1.5 could be achieved by using suspended Si nanowires.

## 2. Analysis and Discussion

In this paper, we consider several typical Si nanophotonic waveguides operating at 1550 nm. The involved materials for nanophotonic waveguides include Si, SiO_2_, and gas (or liquid), whose refractive indices are assumed to be 3.455, 1.445, and 1.0 (or 1.33), respectively. In this paper, we assume that the buffer layer in various SOI optical waveguides considered here is thick enough to make the substrate leakage negligible. For example, the thickness of the SiO_2_ buffer layer is 3 μm in the following calculation.

### 2.1. SOI Nanowires

**Figure 1:**
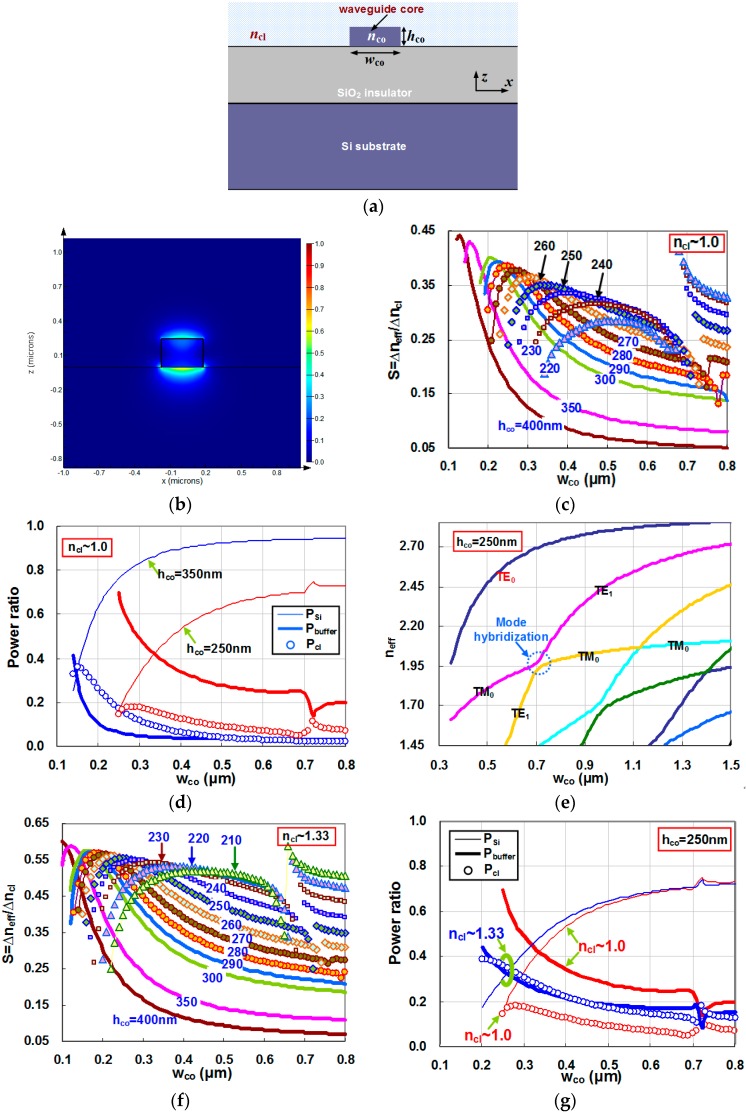
(**a**) The cross section of a SOI nanowire; (**b**) the calculated field distribution of the TM fundamental mode for a Silicon nanowire waveguide with h_co_ = 250 nm, w_co_ = 350 nm, and n_cl_ = 1.0; (**c**) the calculated waveguide sensitivity as the core width varies when the upper cladding is filled with gas (n ~ 1.0); (**d**) the power confinement ratio in the regions of core (P_co_), upper-cladding (P_cl_), and buffer (P_buffer_); (**e**) the effective indices of the eigenmodes for a SOI nanowire with h_co_ = 250 nm; (**f**) the calculated sensitivity as the core width varies when the upper cladding is filled with liquid (n ~ 1.33); and (**g**) the power ratio in the regions of core (P_co_), upper-cladding (P_cl_), and buffer (Pbuffer) when h_co_ = 250 nm.

In this part, we consider a SOI nanowire, which has a Si core and a SiO_2_ buffer layer, as shown in Figure 1a, which is the most popular one used for optical sensing [1]. The upper-cladding is the sample to be measured (*i.e.*, n_s_ = n_cl_), which is a gas (n_s_ = n_cl_ ~ 1) or liquid (n_s_ = n_cl_ ~ 1.33). TM polarization is considered in the following calculation. Figure 1b shows the calculated field distribution of the TM fundamental mode for a Silicon nanowire waveguide with h_co_ = 250 nm, w_co_ = 350 nm , n_cl_ = 1.0 as an example.

Figure 1c shows the calculated sensitivity as the core width w_co_ varies from 0.1 μm to 0.8 μm when the upper-cladding is filled with gas (n ~ 1.0). The Si core layer is 220, 230, 240, 250, 260, 270, 280, 290, 300, 350, and 400 nm thick, respectively. For the case with a relatively thick core (e.g., h_co_ ≥ 350 nm), the sensitivity is low, especially when the core width is large. This is because most power of the fundamental mode is confined in the core region while the evanescent field is very small, as shown in Figure 1d, where P_co_, P_cl_, and P_buffer_ are the power confinement ratio in the regions of core, upper-cladding, and buffer, respectively. When the core width decreases, the confinement becomes weaker and more evanescent field penetrates to the upper-cladding (see Figure 1d). The sensitivity consequently increases, as shown in Figure 1c.

On the other hand, one should note that the present waveguide is asymmetrical in the vertical direction, *i.e.*, the upper-cladding (gas) has a lower refractive index than the buffer layer (SiO_2_). Therefore, when the core becomes very narrow, the optical waveguide cannot confine the optical field well and less power is confined in the upper-cladding region, while more power moves to the SiO_2_ buffer region (see Figure 1d). For example, when w_co_ = 140 nm, the power ratios in the regions of Si core, SiO_2_ buffer, and gas upper-cladding are 25.4%, 41.6%, and 32.9%, respectively. Thus, the sensitivity becomes smaller as the core width decreases further. This explains the existence of an optimal core width w_opt_ for a maximal sensitivity when the core width varies in Figure 1c. For example, when h_co_ = 400 nm, the maximal sensitivity S_max_ = 0.441 when choosing the optimal core width w_opt_ = 130 nm. However, the sensitivity decreases significantly when the core width deviates from the optimal value. Furthermore, such a SOI nanowire with a high aspect ratio is not a good option when considering to the fabrication (e.g., the etching process) and the scattering loss. When choosing a thinner SOI nanowire, there is also an optimal core width w_opt_ for maximal sensitivity. A significant decrease of the sensitivity is also observed when choosing a core width much smaller than the optimal width w_opt_. And the sensitivity around w = w_opt_ is less dependent on the core width in comparison to the case with a large thickness (e.g., h_co_ = 400 nm). Therefore, one could achieve high sensitivity when the core width varies in a large range around w_opt_.

From Figure 1c, we also observe that the sensitivity curve for thin SOI nanowires is discontinuous as the core width decreases from 0.8 μm to 0.6 μm. This is due to the mode hybridization in that width range. In order to explain this, Figure 1d shows the effective index of an SOI nanowire with h_co_ = 250 nm as the core width varies. From this figure, it can be seen that there is a region (see the circle in Figure 1e) around w_co_ = 700 nm, where the TM fundamental mode (TM_0_) and the first-order mode of TE polarization (TE_1_) are hybridized. Therefore, when we consider the sensitivity of TM_0_, a jump appears, as shown in Figure 1c. As can be seen, higher sensitivity S_WG_ can be achieved when choosing the waveguide width around the mode hybridization region. However, when higher-order modes are involved, there might be some undesired multimode effect. For example, when using a microring-resonator sensor, more resonance peaks will appear and the Q-factor will degrade. Therefore, we still focus on the singlemode silicon optical waveguides for optical sensing. Here we also calculate the sensitivity for SOI nanowires with a liquid cladding (n ~ 1.33), which is very popular for biosensing. Figure 1f shows the calculated sensitivity, which is similar to the case for the gas sensing (see Figure 1c). When there is a liquid cladding, the optical waveguide becomes less asymmetrical because the liquid index is closer to that of the SiO_2_ buffer layer. Therefore, less power is confined in the SiO_2_ buffer region and more power is confined in the upper-cladding region, as shown in Figure 1g. Thus, more evanescent optical fields interact with the sample and consequently the sensitivity of a SOI nanowire becomes higher for liquid sensing than that for gas sensing (see Figure 1c,f).

### 2.2. SOI Nanoslot Waveguides

**Figure 2:**
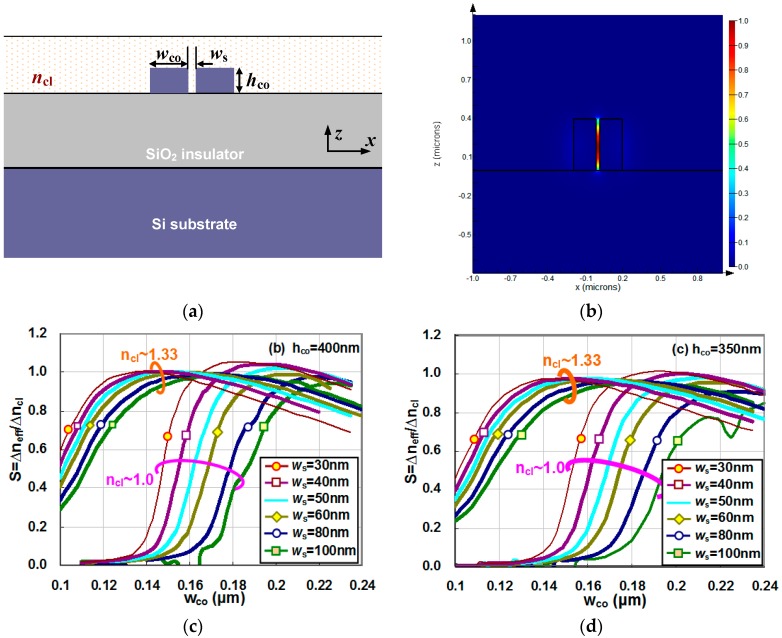

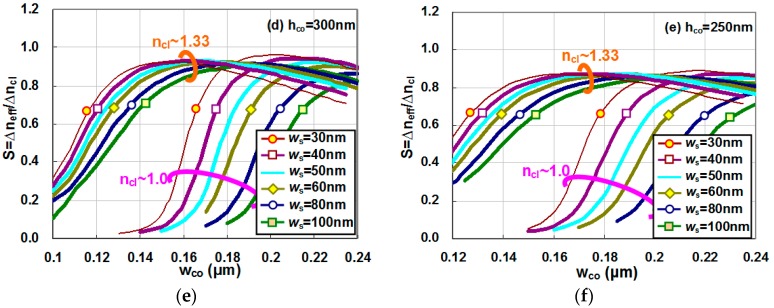
(**a**) The cross section of a nanoslot optical waveguide; and (**b**) the calculated field distribution of the TE fundamental mode for a SOI nanoslot waveguide with h_co_ = 400 nm, w_co_ = 180 nm, and w_s_ = 30 nm as an example. The calculated sensitivity for gas sensing and liquid sensing as the core width varies for the cases with different core heights: (**c**) h_co_ = 400 nm; (**d**) h_co_ = 350 nm; (**e**) h_co_ = 300 nm; and (**f**) h_co_ = 250 nm.

An SOI nanoslot waveguide consists of two Si regions with a nanoslot between them, as shown in Figure 2a. Figure 2b shows the calculated field distribution of the TE fundamental mode for a SOI nanoslot waveguide with h_co_ = 400 nm, w_co_ = 180 nm, and w_s_ = 30 nm as an example. It can be seen that there is a significant field enhancement in the nanoslot region. This field enhancement makes it very attractive for optical sensing when the sample to be measured fills the whole region of upper-cladding (including the nanoslot region).

Figure 2c–f show the calculated waveguide sensitivity for gas sensing as well as liquid sensing as the core width w_co_ varies for the cases of h_co_ = 400, 350, 300, and 250 nm, respectively.The nanoslot width is chosen as 100, 80, 60, 50, 40, and 30 nm for any given core height h_co_. In Figure 2c–f, it can be seen that the sensitivity of a nanoslot waveguide is similar for different values of h_co_ when it is covered by gas or liquid. For a nanoslot waveguide with a given slot width w_s_ and core height h_co_, the sensitivity increases rapidly and then decreases slightly as the core width varies in the range from 0.12 μm to 0.24 μm. It can be seen that there exists an optimal core width w_opt_ for obtaining a maximal sensitivity S_max_ for both cases based on claddings of gas and liquids.

When the core width becomes small (e.g., w_co_ = 0.1 μm), the optical confinement is very weak and the power at the under-cladding becomes dominant due to the vertical asymmetry, especially in the case with a gas cladding. In this case, the optical waveguide become insensitive to the index change of the upper-cladding. In contrast, when there is a liquid cladding, the vertical asymmetry is alleviated and consequently the sensitivity is still relatively high even when the Si core width is reduced to 0.1 μm. On the other hand, when the core width becomes larger (e.g., 0.24 μm), the optical confinement becomes stronger and more power is confined in the Si core region, and consequently the field enhancement in the nanoslot is significantly limited. Therefore, the sensitivity of the nanoslot optical waveguide decreases as the core width increases.

We also note that a nanoslot waveguide with a larger core height gives a higher maximal sensitivity, which could be larger than that of a free-space beam (S = 1). This is because a larger optical field overlaps with the upper-cladding when the core height increases. In contrast, when the core height turns small, more power moves down to the SiO_2_ under-cladding because of the asymmetry in the vertical direction. From Figure 2c–f and Figure 1b, it can be seen that nanoslot waveguides have a much higher sensitivity than conventional SOI nanowires.

### 2.3. Suspended Si Nanowires

**Figure 3:**
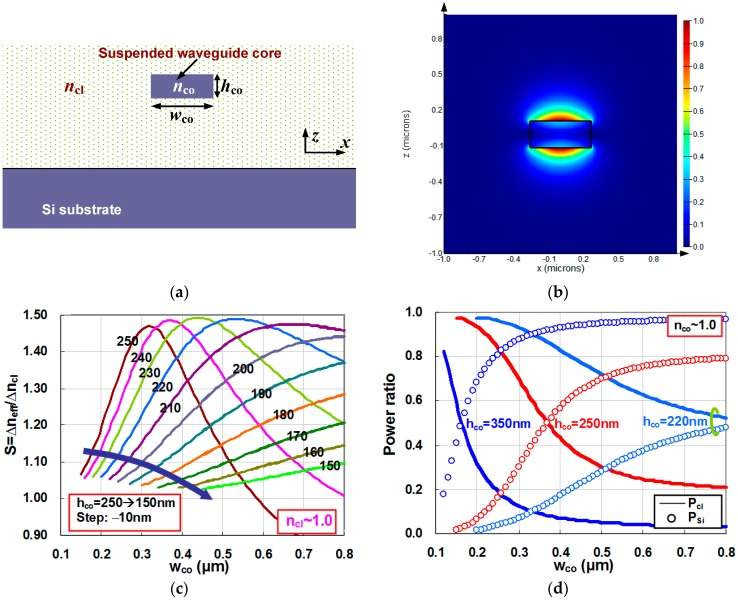

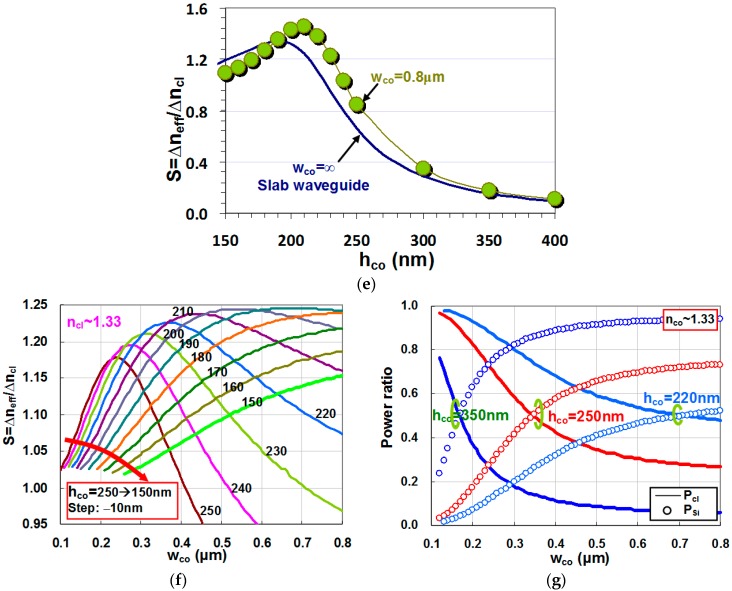
(**a**) The cross section of a suspended Si nanowire; (**b**) the calculated field distribution of the TM fundamental mode for a suspended Si nanowire waveguide with h_co_ = 220 nm, w_co_ = 525 nm, and n_cl_ = 1.0; (**c**) the sensitivity of a suspended Si nanowire for gas sensing (ncl ~ 1.0); (**d**) the power confinement ratio in a suspended Si nanowire for gas sensing; (**e**) the calculated sensitivities for a suspended Si nanowire with w_co_ = 0.8 μm and w_co_ = ∞ (slab waveguide); (**f**) the sensitivity of a suspended Si nanowire for liquid sensing (n_cl_ ~ 1.33); and (**g**) the power confinement ratio in a suspended Si nanowire for liquid sensing.

In this part, we give an analysis for a suspended Si nanowire, which includes a Si core surrounded by the sample, as shown in Figure 3a. Such a suspended waveguide could be achieved with supported arms [21,22]. Figure 3b shows the calculated field distribution of the TM fundamental mode for a suspended Si nanowire waveguide with h_co_ = 220 nm, w_co_ = 525 nm, and n_cl_ = 1.0 as an example. Figure 3c shows the calculated sensitivity for a suspended Si nanowire with different core heights h_co_ as the core width varies from 0.1 μm to 0.8 μm. From this figure, it can be seen that the sensitivity is slightly higher than 1.0 (*i.e.*, the sensitivity of free space) when the core height is small (e.g., 150 nm). This is because the power confined in the Si core is very small while most power of the guided mode locates at the surrounded media, which is very similar to the free space case.

When the core height becomes larger, a significant sensitivity enhancement is observed. For a suspended Si nanowire with a fixed core height (e.g., h_co_ = 100 ~ 200 nm), the sensitivity increases monotonously when the core width ranges from 0.1 μm to 0.8 μm. When increasing the core height further (e.g., h_co_ = 250, 240, 230, 220, and 210 nm), one sees that in the range of 0.1 μm < w_co_ < 0.8 μm there exists an optimal core width w_co_opt_ (which gives a maximal sensitivity S_max_ of >1.0).

In order to explain this, one can use the formula for the sensitivity of a three-layer slab optical waveguide given in [15], *i.e.*,

(1) $$S=\frac{n_{\mathrm{eff}}}{n_{\mathrm{cl}}}\left(2-\frac{n_{\mathrm{cl}}^{2}}{n_{\mathrm{eff}}^{2}}\right)\frac{P_{\mathrm{cl}}}{P_{\mathrm{tot}}}$$

where n_eff_ is the effective index, n_cl_ is the index of the cladding, and P_cl_/P_tot_ is the power confinement ratio in the cladding region. In Figure 3d, we show the power confinement ratio in the regions of core and cladding. In this figure, one sees that the power confinement ratio in the cladding region increases monotonously as the core width decreases, which is easy to understand. Intuitively speaking, one expects to have a higher sensitivity when more power is confined in the cladding region. However, according to Equation (1), the sensitivity depends not only on the ratio of P_cl_/P_tot_ but also the ratio of n_eff_/n_cl_. When the core width decreases, the ratio P_cl_/P_tot_ increases while the ratio n_eff_/n_cl_ decreases, which causes an optimal core width for obtaining a maximal sensitivity.

One can imagine that a wide suspended waveguide should have similar result with a slab waveguide. Figure 3e gives a comparison between the calculated sensitivities for a suspended Si nanowire with w_co_ = 0.8 μm and w_co_ = ∞ (slab waveguide). Here, the results for the slab waveguide (w_co_ = ∞) is calculated with Equation (1). It can be seen that these results are quite similar and there is an optimal core height h_co_ around h_co_ = 220 nm for the maximal sensitivity. For the suspended waveguide with a given core height h_co_, Figure 3c shows that the optimal core width for the maximal sensitivities S_max_ are slightly dependent on the core height. One should choose h_co_ = 220 nm to maximize the sensitivity and the corresponding maximal sensitivity S_max_ is close to 1.5, which is much higher than 1.0 (which is the sensitivity in a free space system). This high sensitivity is due to the high index contrast between the core and the surrounding media (see Equation (1)). The design of h_co_ = 220 nm also has a large tolerance to the variation of the core width. Even when the core width deviates from its optimal value w_co_opt_ (= 0.525 μm), the sensitivity does not decrease much. In comparison with the sensitivity for a SOI nanoslot waveguide (shown in Figure 2c–f), the suspended Si nanowire could provide a much higher sensitivity even though it does not have a field enhancement in the low-index region. Such a significant sensitivity enhancement in a suspended Si nanowire makes it very attractive for the application of optical sensing, especially since it is also relatively easy to fabricate.

Figure 3f shows the sensitivity of a suspended Si nanowire for liquid sensing (*i.e.*, n_cl_ ~ 1.33). Figure 3g shows the power confinement ratios in the regions of core and cladding. It can be seen that the results are similar to those for gas sensing (*i.e.*, n_cl_ ~ 1.0). The power ratio in the cladding region is higher because of the lower index-contrast. However, the maximal sensitivity is lower when there is a higher cladding index, as predicted in Equation (1).

### 2.4. Nanofibers

**Figure 4:**
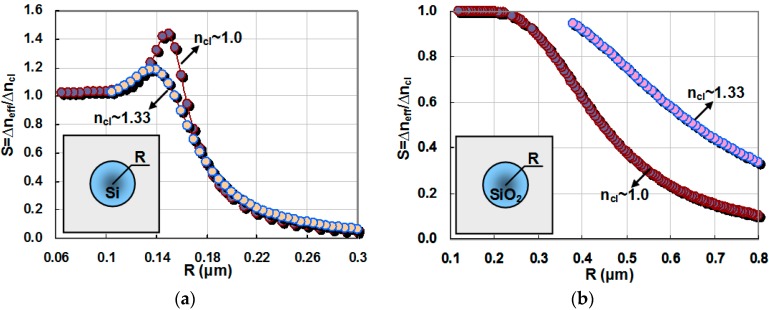
The sensitivity for a nanofiber with: (**a**) Si core; and (**b**) SiO_2_ core.

Nanofibers are another candidate for the use as a suspended waveguide for optical sensing. Since Si is considered for the core of the planar nanophotonic waveguides in the analysis above, we also choose nanofibers based on Si. Figure 4a shows the calculated waveguide sensitivity for a Si nanofiber with gas cladding as the core radius R varies from 0.06 μm to 0.3 μm. The inset shows the cross section of the Si nanofiber. From this figure, one sees that there is an optimal core radius giving a maximal sensitivity, which is similar to the result for suspended Si nanowires. This could be explained as follows: when the core radius is small, the optical confinement is very weak and most power of the guided mode is in the cladding. In this case, the nanofiber behaves like in free space sensing. When the core radius becomes large, the optical confinement becomes very strong. Consequently the evanescent field is very small, which reduces the sensitivity. One might see it in Figure 4a, when it is used for gas sensing, the optimal core radius is about 0.15 μm and the maximal sensitivity S_max_ is about 1.43, which is slightly lower than that of an optimized suspended Si nanowire (see Figure 3b) In contrast, for the case of liquid sensing, the optimal core radius is about 0.14 μm and the maximal sensitivity S_max_ is about 1.2.

When one chooses the optimal core radius for the highest sensitivity, care must be taken because of the strong tolerance upon the core radius dimension. For example, when the core radius varies from the optimal value (*i.e.*, 150 nm) to 165 nm, the sensitivity decreases from 1.43 to 0.943 and one has ∆S/∆R = 32.5/μm (here ∆R is the radius variation and ∆S is the sensitivity decrease). This indicates that one should control the diameter of the nanofiber very carefully in order to achieve the maximal sensitivity. In contrast, the suspended Si nanowire is more tolerant to the lateral dimension variation ∆w_co_. For example, when the core width w_co_ of a suspended waveguide (with h_co_ = 220 nm) varies from the optimal value (*i.e.*, 440 nm) to 455 nm, the sensitivity decreases from 1.477 to 1.475 and one has ∆S/∆w_co_ = 0.13/μm, which is ~240 times less than that of a silicon nanofiber. We note that it is still not easy to fabricate Si nanofibers with the dimension s required for the highest sensitivity. On the other hand, it is well known that nanofibers have been fabricated successfully in the past years using SiO_2_ or polymer [23]. Figure 4b shows the calculated sensitivity of a SiO_2_ nanofiber with air cladding. In this figure, it can be seen that one has a maximal sensitivity close to 1.0 when the core radius of the nanofiber is small. Due to the lower index contrast, one cannot obtain an enhanced waveguide sensitivity of more than 1.0 using SiO_2_ or polymer nanofibers.

## 3. Conclusions

In this paper, a comparative study has been given for the sensitivity of several typical Si nanophotonic waveguides, including SOI nanowires, SOI nanoslot waveguides, suspended Si nanowires, and nanofibers. We have considered two cases of gas sensing (n_cl_ ~ 1.0), and liquid sensing (n_cl_ ~ 1.33). When using SOI nanowires (with a SiO_2_ buffer layer), the sensitivity for liquid sensing is higher than that for gas sensing, due to lower asymmetry in the vertical direction. Our calculations have also shown that it is possible to achieve a waveguide sensitivity higher than that of a free-space sensing (S = 1.0) using SOI nanoslot waveguides, suspended Si nanowires, or Si nanofibers. Among them, the suspended Si nanowires give the highest sensitivity with an optimized core size. One should note that the fabrication process for suspended Si waveguides is more complex than that of standard SOI nanowires. Fortunately such a suspended waveguide can be achieved with supported arms by utilizing additional etching processes to remove the bottom-buffer layer [4,24,25]. When using an optimized suspended Si nanowire for gas sensing, the waveguide sensitivity could be as high as 1.5, which is much higher than that of a SOI nanoslot waveguide. More importantly, the sensitivity of the optimal suspended Si nanowire decreases only very slightly, even when the core width has some variation (e.g., ∆w = ±50 nm). For an optimized Si nanofiber, the sensitivity could be as high as about 1.43, which is close to that of an optimized suspended Si nanowire. However, the tolerance is very small (e.g., the deviation < ±5 nm) and one has to control the diameter of the nanofiber very carefully. The present comparative study has shown that suspended Si nanowire is a good choice to achieve ultra-high waveguide sensitivity.
